# Modified Distributed Bragg Reflectors for Color Stability in InGaN Red Micro-LEDs

**DOI:** 10.3390/nano13040661

**Published:** 2023-02-08

**Authors:** Wen-Chien Miao, Yu-Heng Hong, Fu-He Hsiao, Jun-Da Chen, Hsin Chiang, Chun-Liang Lin, Chien-Chung Lin, Shih-Chen Chen, Hao-Chung Kuo

**Affiliations:** 1 Semiconductor Research Center, Hon Hai Research Institute, Taipei 11492, Taiwan; 2 Department of Electrophysics, College of Science, National Yang Ming Chiao Tung University, Hsinchu 30010, Taiwan; 3 Department of Photonics, Institute of Electro-Optical Engineering, College of Electrical and Computer Engineering, National Yang Ming Chiao Tung University, Hsinchu 30010, Taiwan; 4 Graduate Institute of Photonics and Optoelectronics, National Taiwan University, Taipei 10617, Taiwan

**Keywords:** InGaN, micro-LED, modified DBR, color stability

## Abstract

The monolithic integration of InGaN-based micro-LEDs is being of interest toward developing full-color micro-displays. However, the color stability in InGaN red micro-LED is an issue that needs to be addressed. In this study, the modified distributed Bragg reflectors (DBRs) were designed to reduce the transmission of undesired spectra. The calculated optical properties of the InGaN red micro-LEDs with conventional and modified DBRs have been analyzed, respectively. The CIE 1931 color space and the encoded 8-bit RGB values are exhibited for the quantitative assessment of color stability. The results suggest the modified DBRs can effectively reduce the color shift, paving the way for developing full-color InGaN-based micro-LED displays.

## 1. Introduction

Nowadays, micro-LEDs are deemed as a promising technology for advanced display applications such as augmented reality and virtual reality (AR/VR) as well as visible light communication (VLC) [1,2]. Compared with liquid-crystal and organic LED displays, micro-LED ones have a lot of excellent properties including high brightness, low power consumption, and high resolution [3,4]. In addition, the high stability of its inorganic materials makes micro-LEDs less restrictive and more advantageous for outdoor display applications. Its simple, thin, and compact architecture enables it to achieve a small size or even a flexible display with high pixel density. Despite these excellent characteristics of micro-LEDs, there are challenges including the efficiency degradation by size effect and sidewall effect, high-density defects in the epitaxial layer, mass transfer issues, and other difficult processes. Recently, atomic layer deposition (ALD) passivation has been shown as an effective process to suppress sidewall defects and further increase photoelectric performance [5,6]. Lee et al. fabricated InGaN-based blue LED with an external quantum efficiency (EQE) enhancement of 73.4% by using ALD treatment [7]. Shaping the LED structure is also a valid way to improve device performance, e.g. the tapered structure with a top black matrix can effectively enhance light extraction efficiency and mitigate the color shift of micro-LEDs [8]. To ease the mass-transfer process, the blue or UV LEDs with the color conversion structure such as phosphor or quantum dot (QD) layers are commonly used to achieve a full-color display. The advantages of QDs include a broad absorption spectrum, stable and narrow emission linewidths, and emission of pure and saturated colors which render them more suitable than phosphor for developing full-color micro-LED displays [9,10]. However, QD layers on the top of micro-LEDs still pose big problems for the resolution and uniformity of QD pattern, color conversion efficiency, reliability, and lifetime [11,12,13].

Red, green, and blue (RGB) micro-LEDs are particularly promising for VLC applications since they can be utilized for both displays and multi-channel optical communication simultaneously. The ultra-small LEDs are used in assembling RGB micro-LEDs to achieve full-color displays and allow the brightness of each pixel to be controlled in a dynamic range. In general, RGB micro-LED pixels can be obtained from the mass transfer, InGaN/AlGaN core-shell nanowire heterostructure [14], multi-quantum wells (MQW), or different InGaN-based substrates and monolithically integrated for achieving full-color displays [15]. Among them, InGaN-based blue and green micro-LEDs have attracted a lot of attention due to the efficiency and modulation bandwidth. Hong et al. reported visible-color-tunable LEDs with changeable electroluminescence (EL) color by external electric bias. On GaN nanorod arrays, color-tunable LEDs were created using InGaN/GaN MQW structures and Mg-doped p-GaN overlayers. The EL color varied from red to blue as the applied bias voltage grew from 3.0 V to 10.0 V, covering virtually the whole visible-color spectrum [16]. A full-color micro-LED display with two types of MQWs was reported by Wang et al. which realized a multi-wavelength emission from 450 nm to 620 nm with the increasing injection current [17]. Additionally, monolithically integrating the RGB pixels on the same substrate is also a viable method. Most blue and green micro-LEDs are made of InGaN materials and have been demonstrated without significant loss in their optical performance [18,19]. However, the red micro-LED chips made of AlInGaP are still a big concern due to the dramatic efficiency drops with shrinking size [20]. The efficiency drops have been attributed mostly to the higher surface recombination velocity of AlInGaP LEDs compared to that of InGaN ones. As a result of sidewall damage, AlInGaP LEDs suffer from more Shockley-Read-Hall (SRH) nonradiative recombination, resulting in a drop in the EQE for small-size devices [20]. The efficiency of AlInGaP red micro-LEDs is also very sensitive to temperatures due to the carrier leakage over the quantum barriers and electron-drift-induced reduction in injection efficiency [21]. Hence, extending the emission wavelength of InGaN-based micro-LEDs from blue/green to red appears to be an option for replacing AlInGaP red micro-LEDs due to the size-independent EQE and robust temperature property of InGaN materials. According to a previous paper, InGaN-based RGB micro-LEDs cover 84% of Rec. 2020 [22], and Hartensveld also demonstrated 5 × 5 passive matrices with V-groove color tunable micro-LEDs with the full range of colors from red to blue [23], which are very promising for full-color displays.

**Figure 1:**
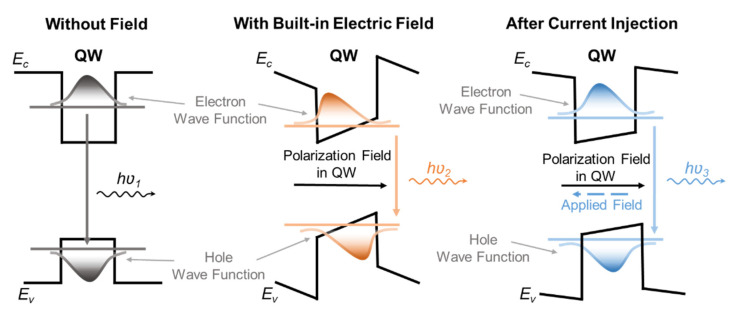
The schematic diagram of the quantum-confined Stark effect (QCSE) in InGaN-based micro-LEDs.

A high-In-content InGaN/GaN QW structure is necessary to achieve red emission. However, the large lattice mismatch between InN and GaN makes the epitaxial process more challenging since a high number of defects and lattice strain would be generated during the growth of high-In-content QWs. Also, the performance of InGaN red micro-LEDs severely suffers from the quantum-confined Stark effect (QCSE), which leads to a low external quantum efficiency (EQE) and the blue shift of emission wavelength as increasing the injected current [24,25,26]. (Figure 1) With electric field applied perpendicular to the quantum well (QW) layer, electrons and holes are separated toward opposite sides of the layer, resulting in a lower radiative recombination rate, and a corresponding Stark shift in the excitonic absorption [14]. Masui, H. et al. reported the electroluminescence (EL) intensities of InGaN-based LED soared because of forward current injection. The emission peak shifted towards higher energy owing to the band-filling and screening effect of injected carriers [27]. The QCSE in InGaN-based micro-LEDs comes from a large polarization-induced electric field, which is also called a built-in electron field, it bends the energy bands in the QW, and reduces the transition energy from the first electron subband to the first hole subband, causing the red-shift of EL emission. When applying the forward bias, excess carriers are injected into the QW and then screen part of the polarization field, leading to a blue shift of spectrum with increasing current density [28]. In previous studies, Lin et al. showed a 9 nm blue shift of EL emission in a 2 × 2 green micro-LED array [29] and Lan et al. also found a slight blue shift both in single-QW and triple-QW GaN-based blue micro-LEDs [30]. 

The blue shifts of EL emission in the InGaN-based blue and green micro-LEDs were not enough to significantly affect the color gamut, but the QCSE in the red ones can be more critical. Although micro-LEDs are driven at low current densities for most microdisplays, some applications such as light engines in projection systems and head-mounted displays, which require high brightness levels, need micro-LEDs to be operated at high current densities. The blue shift caused by QCSE will be non-negligible under high operating current densities and will seriously affect the user experience. To address these issues, several approaches have been found to suppress the QCSE of InGaN red micro-LEDs and enhance the device performance, including growth on semipolar or polar surfaces, use of the patterned sapphire substrate (PSS), use of nanowire or nanocolumn structures and use of superlattice as a strain relaxed layer [31]. Horng et al. studied the relationship between emission wavelength, chip sizes, and injection current densities. As the size of the micro-LED decreased, the blue shift of the emission wavelength became more obvious [32]. Iida et al. demonstrated an epitaxy structure of InGaN red LEDs with a peak wavelength of 633 nm and a blue shift of 40 nm [33], and Zhang et al. also reported a LED device with optimized QW structure and showed a peak wavelength of 608 nm and a blue shift of around 30 nm [34]. Zhuang et al. demonstrated a 47 × 47 µm^2^ InGaN amber micro-LED with a peak wavelength of 606 nm and a 33 nm blue shift [35]. Even though there were several studies of InGaN red micro-LEDs, the EQE and the blue shift phenomenon of the device still have much room for improvement. In our previous work published in 2022 [36], a 25 µm-sized micro-LED was grown on a c-plane patterned sapphire substrate (PSS) by metal-organic vapor-phase epitaxy (MOVPE). The mainly epitaxial structures include an undoped-GaN layer to reduce the residual stress, 15 pairs of GaN/In_0.08_Ga_0.92_N superlattices (SL) layers, an InGaN blue single-quantum well (SQW), an InGaN red double-quantum well (DQWs) with a high indium content, a barrier layer, and contact layers. Furthermore, a thick Al_2_O_3_ layer was deposited by ALD as a passivation layer which helps to repair surface damage caused by dry etching to enhance the performance of the device. Circular-shaped active areas and electrodes were designed to improve current spreading and light extraction efficiency. Although the SL layers improved the quality of the following InGaN QW and released the stress in the structure, thereby mitigating the effect of QCSE, the EL spectra still showed a blue shift of 38 nm, which is much larger than that in InGaN-based blue and green micro-LEDs. As current density increased, the shifted EL emission from 652 nm to 614 nm and the increased full width at half maximum (FWHM) from 48 nm to 64 nm were found. It could seriously affect the color stability of displays and distort the rendered image. To solve this problem, a promising way is to add a color filter at the top of the micro-LEDs to reduce the transmission of undesired spectra. However, the traditional color filter is not suitable for micro-LEDs since its bulky size and non-negligible energy consumption. Therefore, a color filter with a size in the micron range, low absorption in the visible band, and moderate process cost is needed.

**Figure 2:**
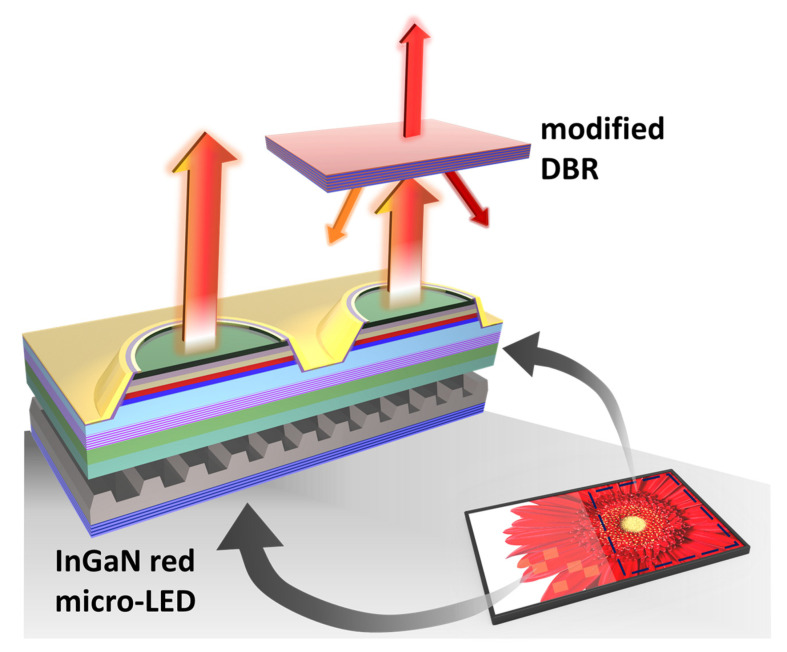
The illustration of the modified distributed Bragg reflector for color stability in InGaN red micro-LED.

A distributed Bragg reflector (DBR) is a reflector constructed with multiple layers of alternating materials with varying refractive indices. In the multilayer structure with a periodic high-low contrast in the optical index of refraction, when the thicknesses of the individual layers equal to a quarter of the wavelength, the partial reflections at each interface combined with constructive interference and the multilayers act as a high-quality reflector with a tunable reflectivity and bandwidth. The DBR technology was first demonstrated in the 1940s and has been commonly used for vertical cavity surface emitting lasers [37,38,39,40,41,42]. The DBRs can achieve near-unity reflectance at the selected wavelengths. Moreover, the high reflectance in the stop band of DBRs can be properly designed as a color filter for optical devices [43,44]. Along with the development of LED display technology, the improved performance of LED devices with DBR structures has attracted a lot of attention. Recent studies have shown that the DBR structure can not only improve light extraction efficiency but also serve as a strain-released layer for InGaN-based micro-LEDs [45,46,47,48]. Although the DBR structures are often used as a mirror, the degree of freedom in reflective wavelengths and the high contrast between reflectance and transmittance is advantageous for filters. Considering the chip size, the difficulty of processing, and the spectrum purity after the filter, a promising way to solve the problem of color shifting is to add a DBR as a color filter on the micro-LED which can completely block the shifting wavelengths. In this study, based on the experimental results of InGaN red micro-LED in our previous work [36], the modified DBRs were proposed as color filters of the InGaN red micro-LEDs (Figure 2).

## 2. Experiments

The DBR structures were designed in RSoft Photonics computer-aided design (CAD) Suite (version 2021.09-1, Synopsys, Mountain View, CA, USA) and the optical simulations were carried out by using 2D diffraction mode. The material parameters for building the structure along with the dispersive complex-valued refractive index models were acquired from the built-in material library of RSoft. The background index used in the simulation is 1. For the quantitative assessment of the color stability in InGaN-based red micro-LEDs with different DBR structures, the calculated spectra were converted to the tristimulus values in CIE 1931 color space by MATLAB (academic, R2021a). 

### 2.1. Conventional DBRs

For conventional DBR structures, the wavelength (λ) of incident light determines the thicknesses of the high- and low- refractive index (n) layers by Equation (1):

Thickness = λ/4n (1)

When the refractive index difference between the two materials becomes larger, a higher and broader reflection spectrum occurs. For designing a long pass filter for InGaN red micro-LED, the central wavelength of the reflection spectrum should be less than 600 nm so that the edge of the reflection spectrum can be located at a proper wavelength to block the light caused by blue shift.

**Figure 3:**
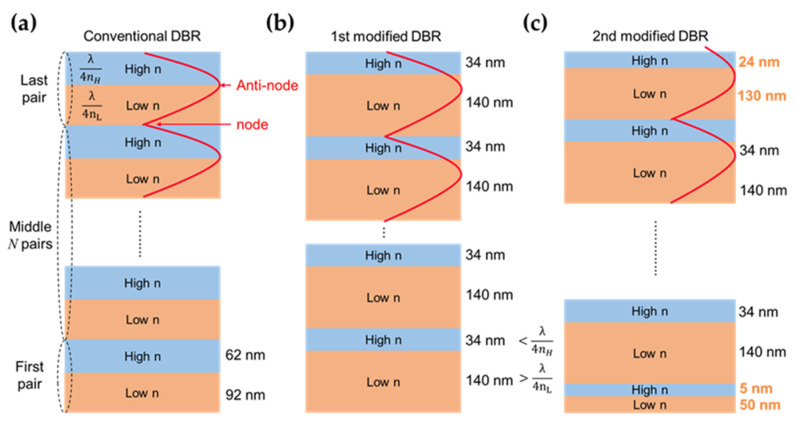
Schematic illustrations for (**a**) conventional DBR, (**b**) 1st modified DBR, and (**c**) 2nd modified DBR structures.

In this study, TiO_2_ and SiO_2_ were adopted as the high-n and low-n materials for the DBR structures, respectively. The refractive index and extinction coefficients for the materials were obtained from the library of the RSoft software together with the report from Sarkar et al. [49,50]. In the wavelength range of visible light, refractive index of TiO_2_ is above 2.5, and that of SiO_2_ is about 1.5. Furthermore, the strong absorption only appears at 300 nm to 400 nm due to the high extinction coefficient of TiO_2_ in that region, which is far from the red light region and has little effect on the transmission spectra. This means that the energy loss caused by material absorption of DBR in the red light region is almost negligible. Hence, the DBR with the central wavelength of 540 nm was designed to block wavelengths below 620 nm, the thickness of TiO_2_ film and SiO_2_ film were 62 nm and 92 nm, respectively (Figure 3a). Based on the transfer matrix method (TMM) and geometrical optics [51], the transmittance and reflectance spectra of the DBR structures consisting of TiO_2_/SiO_2_ pairs were calculated.

### 2.2. Modified DBRs

In general, the strong spectral ripples of conventional DBR are associate with the high-order interference in the high-n/low-n structure [52]. These ripples are detrimental to micro-LED display performance such as luminance, efficiency, or the vividness of colors. To reduce or even annihilate the undesired ripples, the modified DBR structures were proposed by Lin et al. in 2021 [52]. There are two architectures labeled with 1st modified DBR and 2nd modified DBR shown in Figure 3b,c. To maximize the reflectance of the DBRs at the blocking region, the electric field should be minimized at the exiting interface. The node appears at the interface between two adjacent pairs where the electric field should be closed to zero, and the antinode would occur at the high-n/low-n interface in a pair. The 1st modified DBR structure, with the standing wave peak deviated from the interface, can weaken high-order interference and remain the same resonant wavelength. Based on the conventional structure, the thicknesses of the high-n and low-n layers in 1st modified DBR are relatively smaller and larger than the quarter of the central wavelength. For the 1st modified DBR, the thickness of TiO_2_ and SiO_2_ was 34 nm and 140 nm, respectively (Figure 3b).

Ideally, a flat transmission spectrum with a transmittance over 90% in the long wavelength range is desired. Although the strong ripples can be reduced in the 1st modified DBR structure, the advanced design that can eliminate the high-order modes of constructive interference was proposed as 2nd modified DBR. The thicknesses of the first and last pairs were adjusted respectively for eliminating the ripples. For the optimized structure, the first pair of SiO_2_/TiO_2_ (50 nm/5 nm), the last pair of SiO_2_/TiO_2_ (130 nm/24 nm) were designed (Figure 3c).

## 3. Results and Discussion

**Figure 4:**
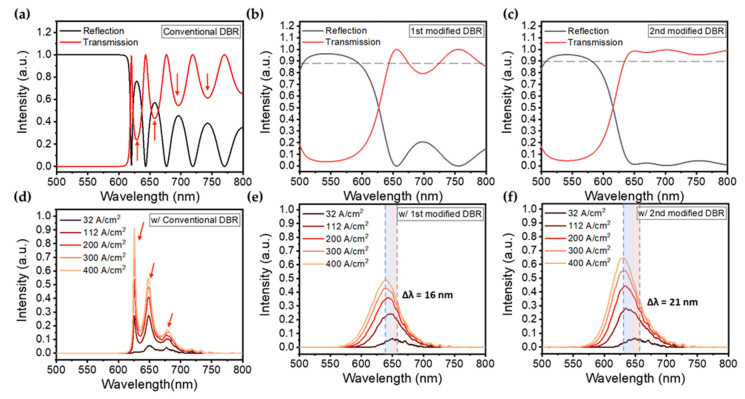
Calculated transmittance and reflectance spectra of (**a**) conventional DBR structure, (**b**) the 1st modified DBR structure, and (**c**) the 2nd modified DBR structure, the dotted line represents the transmittance of 90%. The electroluminescence spectra of the InGaN red micro-LEDs (**d**) with conventional DBR, (**e**) with the 1st modified DBR and (**f**) with the 2nd modified DBR. Red arrows indicate the center of ripples and multi-peaks, and the colored region shows the range of blue shift.

The calculated transmittance and reflectance spectra of the conventional and two modified DBR structures are shown in Figure 4a–c, and their convolutions with the EL spectra of the InGaN red micro-LED in our previous work (Figure 4d–f). The conventional DBR was designed with 18 pairs of TiO_2_/SiO_2_ to achieve a sharp edge of the transmission spectrum. As Figure 4a shown, the conventional DBR can highly reflect the wavelengths from 500 nm to 630 nm, but generate the undesired ripples above 630 nm. The ripples are respectively centered at 630, 660, 700, and 740 nm, which can be detrimental to the EL emission in the InGaN red micro-LED. The calculated EL spectra of the InGaN red micro-LED with the conventional DBR are shown in Figure 4d, which exhibit the multi-peaks due to the blocking from the ripples. Although the wavelengths below 620 nm can be totally blocked by the conventional DBR structure, the existence of ripples could seriously affect the color stabilization. In the 1st modified DBR structure, the modification was based on the conventional configuration. (Figure 3b) As a result, only a small ripple appears at 700 nm, as shown in Figure 4b. Although the edge of the spectrum is not as sharp as the conventional configuration, a high transmittance of over 90% can be remained with only 8 pairs of SiO_2_/TiO_2_. The edge of the high reflection region is located nearby 650 nm, and the transition region where the reflectance dropped from 0.9 to 0.1 is wider than that of the conventional DBR, leading to the smooth Gaussian-like spectra (Figure 4e). The blue shift of EL emission peak can be observed as increasing current density.

The calculated results of the 2nd modified DBR are shown in Figure 4c,f. It is obvious that the ripples are effectively suppressed due to the thickness optimization of the modified structure. The edge of the high reflection region is slightly blue-shifted compared to that of the 1st modified DBR. As a result, the higher intensity of EL emission can be observed due to the high transmission over 650 nm.

**Figure 5:**
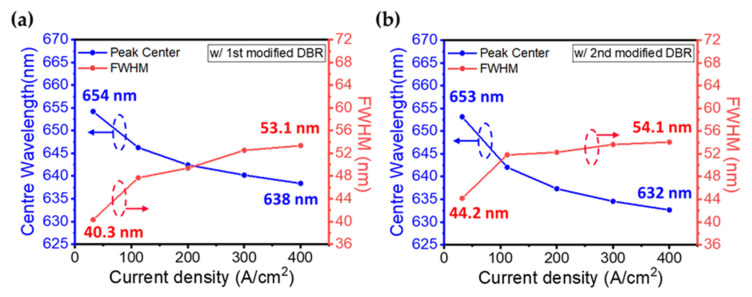
Centre wavelength and FWHM of EL emission as a function of current density for the InGaN red micro-LEDs (**a**) with the 1st modified DBR and (**b**) with the 2nd modified DBR.

The central wavelength and FWHM of the EL mission as a function of current density for the InGaN red micro-LEDs with 1st and 2nd modified DBRs are shown in Figure 5. As Figure 5a shown, the peak position of the EL emission shows a blue shift of 16 nm which is much smaller than that in our previous work [36]. The FWHM of EL emission is broadened from 40 nm to 53 nm when the current density increased from 32 A/cm^2^ to 400 A/cm^2^. In Figure 5b, a blue shift of 21 nm for the EL mission of InGaN red micro-LED with the 2nd modified DBRs can be found. Compared to the 1st modified DBR, the peak shift is 5 nm larger, but the intensity of EL emission is improved by 40% (Figure 4b,c) that is important for color purity.

For the quantitative assessment of color stability, we converted the calculated spectrum into the tristimulus values in CIE 1931 color space. The tristimulus values were calculated by the integration of the EL spectra and the CIE standard colorimetric observer curve based on the distribution of cones in the eye [53,54]. The CIE xy chromaticity is specified by the two derived parameters x and y, two of the three normalized values being functions of all three tristimulus values X, Y, and Z. By converting the spectra into the chromaticity diagram, the color stability of the InGaN red micro-LEDs with the DBR structures can be quantitatively analyzed.

**Figure 6:**
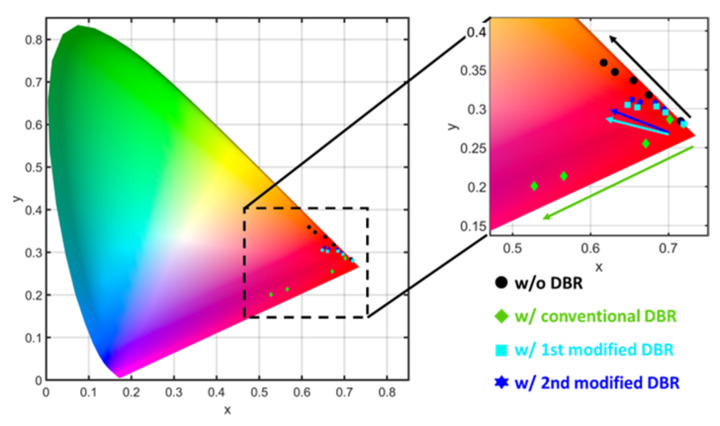
The CIE 1931 color space for the InGaN red micro-LEDs with different DBRs.

The normalized tristimulus values and the CIE 1931 xy chromaticity were calculated and plotted (Figure 6) [55]. The CIE 1931 chromaticity diagram for the InGaN red micro-LED with DBR structures operated at current densities are displayed. The calculated results suggest that all tristimulus values are in the red-light region. At the lowest current density of 32 A/cm^2^, each value is located at the corner of the color map. With the current density increased, the color coordinates move from the corner to the inner part, narrowing down the range of the color gamut. In Figure 6, the data for both modified DBR ones are respectively labeled with blue star and cyan square, suggesting a less shift compared with the conventional DBR and bare ones (w/o DBR) [52]. In order to analyze the difference among these designs, the distances of color shift were calculated in detail. For the InGaN red micro-LED without DBR, the color coordinate shifts from (0.7156, 0.2843) to (0.6169, 0.3591) as the current density increased from 32 A/cm^2^ to 400 A/cm^2^. When a conventional DBR acts as a color filter, the coordinate shift from (0.7193, 0.2807) to (0.5277, 0.2006). Compared to the bare one, the straight-line distance of the color shift for the conventional DBR increased by 67%, which indicates the negative effect for color stability. The result is reasonable since the spectra of the conventional DBR one show multi peaks (Figure 4d) which can decrease the color purity. The 1st modified DBR one shows the coordinates shifted from (0.7195, 0.2804) to (0.6474, 0.305), a straight-line distance of 0.076 which is 38% less than the bare one. The coordinates for the 2nd modified DBR one shift from (0.7179, 0.2801) to (0.6351, 0.3117) with a straight-line distance of 0.071 that is 42% less than the bare one. Also, this result indicates that the modified DBR design can be used as a novel color filter with a small form factor to effectively improve the color stability in InGaN-based red micro-LEDs.

At the current density of 32 A/cm^2^, the coordinates for different designs are all around (0.71, 0.28) in the CIE color space. While the current density increased to 400 A/cm^2^, the color become amber and magenta for the InGaN red micro-LEDs without and with conventional DBR. In contrast, the colors for the ones with modified DBRs are not significantly changed. To further identify the color difference between both modified DBR designs, the 8-bit encoded RGB values were calculated and listed in Table 1 [56,57].

## 4. Conclusions

In summary, we have developed the modified DBRs to mitigate the blue shift in the InGaN red micro-LEDs. The optical properties of three different DBR designs, including the conventional DBR, the modified DBRs without and with thickness optimization were elaboratively compared. The calculated spectra were converted into the CIE 1931 color space and the 8-bit encoded RGB values for the quantitative assessment of color stability. As a result, the conventional DBR increased the color shift by 67% since the spectral ripples reduced the color purity, leading to the color changed from red to magenta. In contrast, the modified DBRs without and with thickness optimization decreased the color shift of the InGaN red micro-LEDs by 38% and 42%, respectively. It has been demonstrated that the modified DBR structure can effectively improve the color stability, which holds great promise for developing full-color displays of InGaN-based micro-LEDs.

## Figures and Tables

**Table 1:** The calculated x, y value in the CIE 1931 color space and the corresponding RGB color code for the InGaN red micro-LED with different DBRs.

Current Density	Format	w/o DBR	w/Conventional DBR	w/1st Modified DBR	w/2nd Modified DBR
32 A/cm^2^	XYZ ^1^	(0.7156, 0.2843)	(0.7193, 0.2807)	(0.7195, 0.2804)	(0.7179, 0.2821)
	RGB ^2^	[255, 0, 0]	[255, 0, 0]	[255, 0, 0]	[255, 0, 0]
400 A/cm^2^	XYZ ^1^	(0.6169, 0.3591)	(0.5277, 0.2006)	(0.6474, 0.305)	(0.6531, 0.3117)
	RGB ^2^	[255, 78, 0]	[255, 0, 143]	[255, 0, 43]	[255, 0, 25]

^1^ CIE XYZ color space, the format is represented by (x, y). ^2^ 8-bit encoded RGB, the format is represented by [R, G, B].

## Data Availability

The data presented in this study are available on request from the corresponding author.
